# When Two Lives Are at Stake: Sinonasal Adenoid Cystic Carcinoma During Pregnancy

**DOI:** 10.7759/cureus.84927

**Published:** 2025-05-27

**Authors:** Sandra Marie Joe, Deepa Karmali, Vishal V Bhende

**Affiliations:** 1 Obstetrics and Gynaecology, Goa Medical College and Hospital, Bambolim, IND; 2 Paediatric Cardiac Surgery, Bhanubhai and Madhuben Patel Cardiac Centre, Shree Krishna Hospital, Bhaikaka University, Karamsad, IND

**Keywords:** adenoid cystic carcinoma, chemotherapy, head and neck cancer, pregnancy, radiotherapy, rare cancer in pregnancy, sinonasal malignancy

## Abstract

Sinonasal malignancies during pregnancy present unique diagnostic and therapeutic challenges due to their rarity and nonspecific clinical presentation. This case describes a 28-year-old primigravida patient at 32 weeks of gestation, who presented with left-sided nasal obstruction, epistaxis, and facial swelling. Clinical evaluation, imaging, and histopathology confirmed adenoid cystic carcinoma of the left maxillary sinus. Given the advanced disease stage, a multidisciplinary team opted for conservative management until fetal viability was ensured. At 35 weeks and 3 days of gestation, the patient underwent an emergency cesarean section, delivering a healthy neonate. Postpartum, she underwent left total maxillectomy, followed by adjuvant radiotherapy and five cycles of intravenous cisplatin chemotherapy. Long-term follow-up demonstrated stable disease, with minor speech difficulties and adequate oral intake. This case highlights the importance of early recognition, individualized treatment strategies, and multidisciplinary collaboration in managing malignancies during pregnancy, while balancing maternal prognosis and fetal safety. Further research is needed to establish standardized treatment guidelines for rare malignancies in pregnancy.

## Introduction

Sinonasal malignancies are rare tumors, accounting for less than 5% of all head and neck neoplasms, with a total incidence of 0.5-1.0 per 100,000 individuals [1]. Malignancy during pregnancy is uncommon, occurring in approximately 1 in 2,000 pregnancies [2]. The nasal cavity is the most frequently affected site (40% to 50%), followed by the maxillary sinus (30% to 40%) and the ethmoid sinus (10% to 15%) [3]. These malignancies are more common in men, and their initial symptoms are often nonspecific, leading to delayed diagnosis at an advanced stage.

Common presenting symptoms, including periorbital pain, nasal congestion, epistaxis, and unilateral nasal obstruction, often mimic rhinosinusitis, further complicating early detection. Prognostic factors include tumor stage, histological differentiation, and lymph node involvement. Most sinonasal malignancies require surgical resection, with adjuvant radiotherapy and/or chemotherapy. We present a case that highlights the importance of early recognition, accurate diagnosis, and a coordinated management approach during pregnancy.

## Case presentation

This study was approved by the Institutional Ethics Committee of Goa Medical College (Approval No. GMCIEC/2025/07, dated February 25, 2025). Written informed consent was obtained for this case report. The study was conducted in the Department of Obstetrics and Gynaecology at Goa Medical College and Hospital, Bambolim, India.

**Figure 1:**
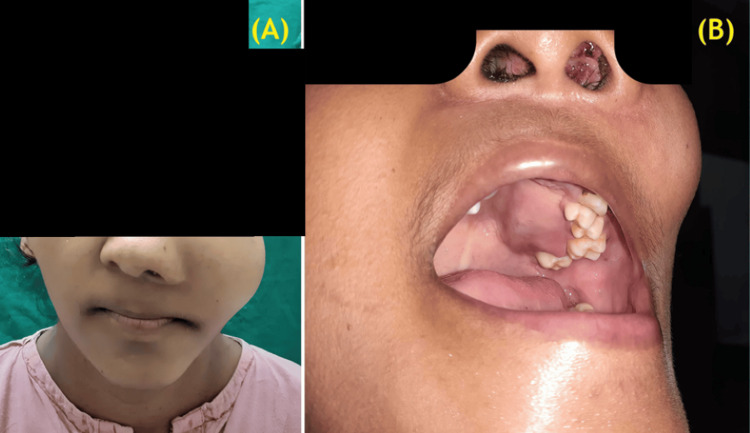
(A) Preoperative image showing facial asymmetry due to left malar enlargement; (B) Intraoral examination revealing tumor-induced palatal expansion. A friable, proliferative mass occupying the left nasal cavity is also observed.

A 28-year-old primigravida at 32 weeks of gestation presented with left-sided nasal obstruction, occasional epistaxis, and gradually increasing swelling over the left cheek for three weeks. She had no obstetric concerns. Fetal growth parameters and antenatal investigations were within reference limits. Clinical examination revealed left malar swelling and a proliferative mass in the left nasal cavity, prompting referral to the Otorhinolaryngology Department for further evaluation (Figure 1).

Direct nasal endoscopy revealed a friable, proliferative mass in the left nasal cavity that bled on contact, along with left malar fullness, proptosis, and palatal bulging. Histopathology and immunohistochemistry confirmed adenoid cystic carcinoma (ACC) of probable salivary origin. Magnetic resonance imaging (MRI) demonstrated a large, heterogeneous mass involving the entire nasal cavity, left maxillary sinus, bilateral ethmoid and sphenoid sinuses, retro-maxillary space, and orbit, with optic nerve compression and extension into the left frontal sinus. A multidisciplinary team formulated a management plan.

At 33 weeks of gestation, antenatal corticosteroids were administered to promote fetal lung maturity. At 35 weeks and 3 days, the patient was admitted with preterm premature rupture of membranes and underwent an emergency lower-segment cesarean section, delivering a live female neonate (2.1 kg, Apgar scores: 8/9). Her postpartum recovery was uneventful. The patient was subsequently transferred to the Otorhinolaryngology Department for further evaluation and intervention.

**Figure 2:**
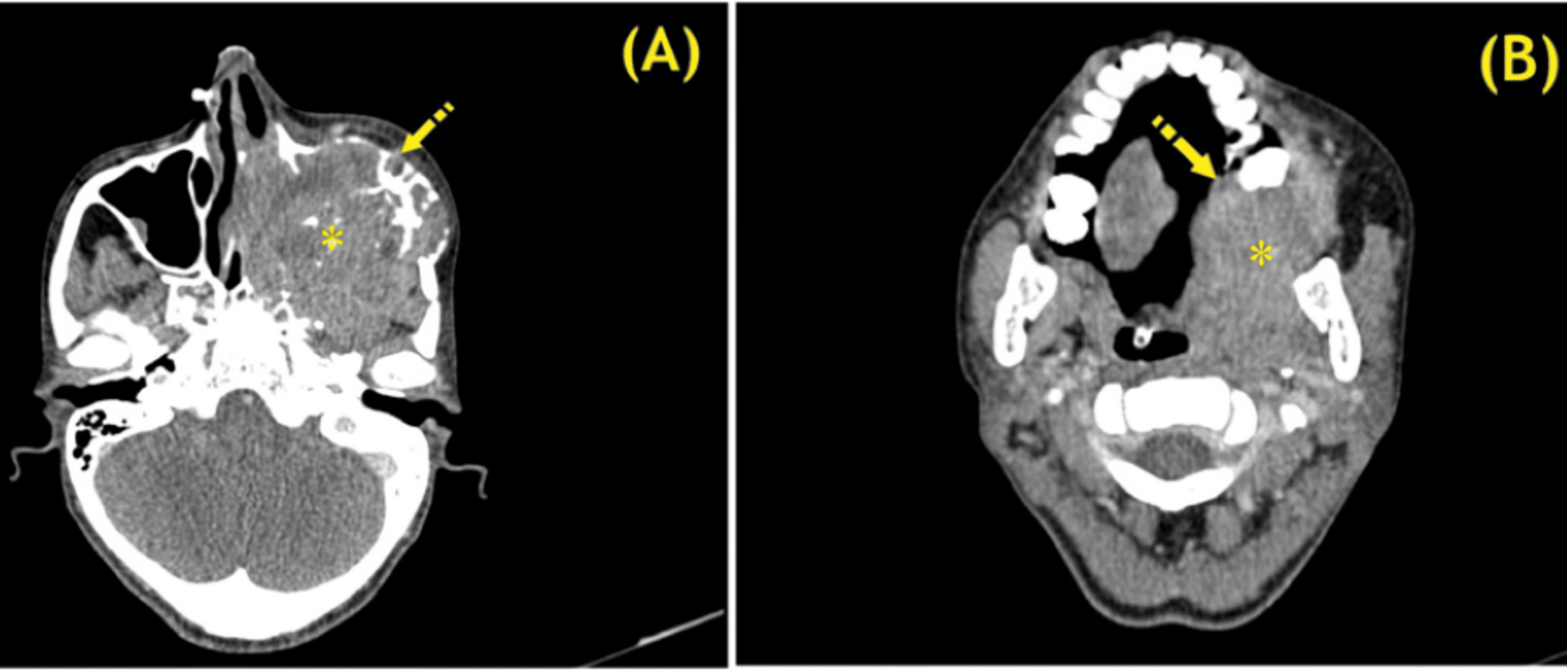
(A) Axial CECT scan of the head and neck showing a large, heterogeneously enhancing mass (asterisk) occupying the nasal cavity and left maxillary sinus, with destruction of the medial wall of the left maxillary sinus, and partial destruction and bowing of the anterior and lateral maxillary sinus walls (arrow); (B) Axial CECT scan showing inferior tumor extension (asterisk), causing dehiscence of the hard palate and intraoral protrusion on the left side (arrow). CECT, contrast-enhanced computed tomography

Contrast-enhanced computed tomography of the paranasal sinuses and MRI of the brain confirmed the extent of the disease, staging the malignancy as ACC of the left maxillary sinus (T4aN0M0, stage IVa; Figure 2).

The patient underwent left total maxillectomy via the Weber-Ferguson approach, with split-thickness skin grafting and temporary placement of an immediate palatal obturator.

Anesthesia and pain management

Surgery was performed under general anesthesia using a cuffed 7.5-Fr endotracheal tube. Postoperatively, the patient was transferred to the intensive care unit (ICU) for gradual weaning from ventilatory support and supportive care. Intraoperatively, she received intravenous (IV) morphine (6 mg). Postoperatively, she was managed with a continuous IV fentanyl infusion (1 mcg/kg/hr) and midazolam infusion (0.2 mg/kg/hr) on the first postoperative day. She was weaned off ventilatory support on the second postoperative day. A total fentanyl bolus dose of 120 mcg/kg was administered, along with IV paracetamol (1 g every eight hours). She was transferred out of the ICU on the third postoperative day.

Surgical procedure

The patient underwent left total maxillectomy via the Weber-Ferguson approach, with split-thickness skin grafting and temporary immediate palatal obturation. Details of the surgical procedure are summarized in Table 1 [4].

**Table 1:** Surgical steps for left maxillectomy Table credit: [4]

Step	Procedure	Description
1	Incision and Flap Elevation	Weber-Ferguson incision made, deepened, and hemostasis secured. Subcutaneous flaps elevated laterally beyond the zygomaticomaxillary suture line.
2	Vascular and Muscle Management	Left facial artery branches identified and ligated. Left masseter muscle released from its zygomatic attachment. Left medial canthal ligament transected.
3	Osteotomies and Tumor Exposure	Osteotomy performed at the zygomaticomaxillary suture. Midline nasal bone osteotomy completed. Maxillary and palatal osteotomies extended to the hard-soft palate junction.
4	Orbital Clearance and Tumor Dissection	Tumor dissected off extraocular muscles inferiorly, noted to be infiltrating the periorbita. Periorbital fat appeared healthy.
5	Tumor Resection	Tumor excised along with the left nasal bone and deviated septum. Specimen separated posteriorly from the pterygoid plate and sent for histopathology.
6	Hemostasis and Skull Base Clearance	Left sphenopalatine artery cauterized. Residual tumor on the posterior wall of the pterygopalatine fossa and skull base excised using a microscope and sent for histopathology.
7	Endoscopic Examination	Sphenoid sinus examined via endoscopy and confirmed to be free of tumor.
8	Reconstruction and Closure	Split-thickness skin graft harvested from the thigh and sutured over the raw area. Defect packed with paraffin gauze. Immediate temporary palatal obturator placed and secured.
9	Surgical Closure	Defect sutured in layers using 4/0 polyglactin for subcutaneous tissue and 4/0 silk for skin closure. Throat pack removed, and oropharyngeal suctioning performed. Ryle’s tube inserted and position confirmed.
10	Postoperative Care	Patient transferred to the intensive care unit for monitoring and supportive care.

**Figure 3:**
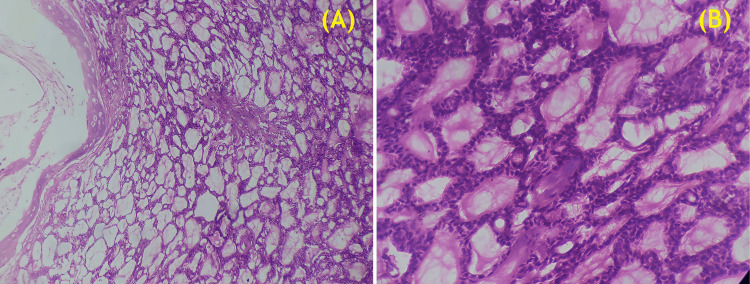
(A) Light microscopy image (hematoxylin and eosin staining, 10× magnification) showing a moderate-grade adenoid cystic carcinoma with a characteristic cribriform growth pattern (scale bar: 200 μm); (B) High-power light microscopy image (hematoxylin and eosin staining, 40× magnification) highlighting the histopathological features of adenoid cystic carcinoma, including basaloid tumor cells with hyperchromatic nuclei and scant cytoplasm (scale bar: 50 μm).

A histopathological examination of the excised specimen confirmed moderate-grade ACC (Figure 3). 

**Figure 4:**
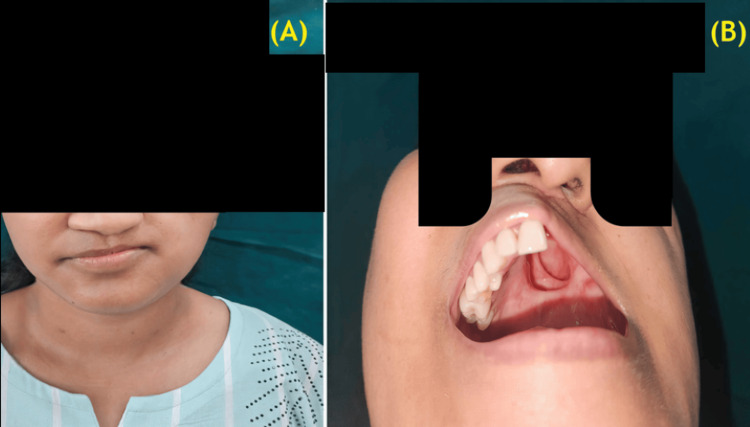
(A) Postoperative photograph, following left hemi-maxillectomy and skin grafting; (B) Intraoral examination, after hemi-maxillectomy.

Two weeks postoperatively, nasal endoscopy was performed. The patient subsequently received radiotherapy. Over time, her condition improved. In coordination with the Oromaxillofacial Surgery Department, she was provided with a palatal obturator prosthesis and dentures. She is currently stable, with minor speech difficulties and adequate oral intake (Figure 4).

Results

Following surgical intervention, the patient was discharged after a two-week hospital stay. During this period, the Ryle’s tube was removed, and a gradual transition from a liquid to a soft diet was achieved. In the initial postoperative phase, she experienced nasal regurgitation, dysphagia, and impaired speech. However, with the fabrication and serial adjustments of a palatal prosthesis and dentures, along with the use of a mouth-opening device, significant improvements were observed in swallowing and speech function.

As part of her oncologic treatment plan, she underwent radiotherapy, receiving 33 fractions of image-guided radiation therapy and intensity-modulated radiation therapy, along with five cycles of chemotherapy with IV cisplatin (40 mg). Over time, her general condition and quality of life progressively improved.

The patient has now completed three years postoperatively. In the first year, follow-up consultations were conducted at three-month intervals, followed by six-month intervals in the second year. From the third year onward, she will continue with annual follow-up assessments. At present, she remains clinically stable, with only minor speech difficulties and adequate oral intake.

## Discussion

Cancer during pregnancy is exceptionally rare, with an incidence of 0.07% to 0.1% of all malignancies [5]. The most frequently reported malignancies in pregnancy include breast cancer, lymphoma, cervical cancer, melanoma, thyroid cancer, ovarian tumors, and brain tumors [5]. Sinonasal malignancies are among the rarest forms of head and neck cancers, accounting for less than 1% of all malignancies [6]. Their occurrence during pregnancy is even more uncommon. The clinical presentation is often nonspecific, with symptoms such as nasal congestion, periorbital pain, unilateral nasal obstruction, and epistaxis, which can mimic benign conditions like rhinosinusitis or pregnancy-related nasal congestion. As a result, delays in diagnosis are common, leading to advanced-stage presentation and complicating treatment options.

Early recognition of sinonasal ACC in pregnant patients requires a high index of clinical suspicion. Certain red flags, such as persistent unilateral nasal obstruction, recurrent or unexplained epistaxis, facial swelling, or localized pain not responding to standard medical therapy, should prompt further evaluation beyond empirical treatment for sinusitis. In such cases, nasal endoscopy serves as a safe and effective first-line tool that does not involve radiation exposure and can help detect suspicious lesions warranting biopsy.

Sinonasal cancers comprise a diverse group of tumors with varying histological types. The most common include squamous cell carcinoma, adenocarcinoma, ACC, sinonasal undifferentiated carcinoma, malignant lymphoma, olfactory neuroblastoma (esthesioneuroblastoma), mucosal melanoma, and soft tissue sarcoma [7]. Squamous cell carcinoma is the most prevalent, accounting for over 50% of cases, primarily arising in the nasal cavity and maxillary sinus. Adenocarcinoma constitutes 10% to 20% of cases, while ACC, a slow-growing neoplasm, represents 5% to 7%. Sinonasal undifferentiated carcinoma, a rare but aggressive entity, occurs in 3% to 5% of cases. Malignant lymphoma, predominantly non-Hodgkin lymphoma, requires a distinct therapeutic approach. Olfactory neuroblastoma, originating in the upper nasal cavity, accounts for 2% to 6%, while mucosal melanoma comprises 4% to 8%. Soft tissue sarcomas, though rare, can also affect this region. Given the variability in biological behavior, early diagnosis and individualized treatment are essential for improving patient outcomes [8].

Sinonasal ACC is an extremely rare entity, with only four previously reported cases in pregnant patients. To the best of our knowledge, this is the fifth reported case of sinonasal ACC presenting with partial upper airway obstruction in pregnancy [9].

Studies have demonstrated a link between the development of sinonasal cancer and the presence of estrogen and progesterone receptors (PRs) in tumor cells [10-12]. ACC is the most common sinonasal tumor with estrogen receptor (ER) expression, suggesting a potential role for hormone therapy in management. However, immunohistochemistry in this case showed the tumor was negative for ER and PR expression, which is associated with a worse prognosis. A study by Luo et al. emphasized the role of hormone therapy in ACC, as 75% of cases were positive for ERα, while 17% did not express ER [10].

Pregnancy complicates oncologic care, as physiological and hormonal changes can influence tumor behavior. Managing cancer in pregnancy requires a multidisciplinary approach to balance maternal prognosis with fetal safety. Treatment decisions depend on gestational age, tumor stage, and fetal risks. The first trimester presents the highest teratogenic risk, making chemotherapy and radiotherapy contraindicated, while surgical interventions require caution. The second trimester offers a safer window for surgery and selected chemotherapies. In the third trimester, maternal stabilization is prioritized, with surgical intervention as needed, while adjuvant therapies are typically postponed until after delivery to prevent neonatal complications [2]. Treatment strategies must be carefully tailored to optimize maternal and fetal outcomes, necessitating close interdisciplinary coordination and individualized decision-making. Surgical resection remains the primary treatment for sinonasal malignancies and is considered the gold standard when feasible. However, pregnancy imposes limitations on the use of radiotherapy and chemotherapy, particularly during the first trimester, due to potential teratogenic effects. In this case, given the advanced stage of malignancy, conservative management was pursued until fetal viability was ensured, after which the patient underwent an emergency preterm cesarean section to facilitate definitive oncologic treatment postpartum. Practitioners should assess each case based on tumor aggressiveness, gestation, and available supportive care. While maternal survival remains paramount, thoughtful deferral may be justified when the window before fetal viability is short, and the tumor demonstrates relatively slow progression.

Existing literature suggests that malignancy during pregnancy increases the risk of preterm labor and preterm premature rupture of membranes [13], as observed in this patient. This may be attributed to an inflammatory response triggered by cancer or its treatment [13]. The decision to administer antenatal corticosteroids at 33 weeks was crucial in promoting fetal lung maturity and reducing the risk of neonatal respiratory distress. Following the diagnosis of sinonasal ACC, enhanced fetal surveillance was done to assess well-being and support the timing of delivery. Serial growth scans, biophysical profiles, and Doppler studies were performed to monitor fetal development and placental function. Non stress tests were also performed periodically to assess heart rate patterns and identify any signs of fetal distress. As no specific prenatal genetic testing was indicated in this case, the obstetric team was actively involved in ensuring comprehensive antenatal care, including readiness for early delivery if maternal condition worsened or fetal compromise was noted.

Postpartum, the patient underwent tumor resection followed by adjuvant radiotherapy and chemotherapy, consistent with standard treatment protocols for sinonasal ACC. Given its propensity for perineural invasion and local recurrence, long-term follow-up is essential to monitor disease progression and assess treatment efficacy.

## Conclusions

The diagnosis of malignancy during pregnancy presents unique diagnostic and therapeutic challenges. Given the rarity of sinonasal malignancies, early recognition and timely intervention are crucial. A multidisciplinary approach is essential to optimize both maternal and fetal outcomes. Surgery remains the primary treatment modality, while radiotherapy and systemic therapy must be carefully tailored to balance oncologic necessity with fetal safety. This case highlights the need for further research and standardized treatment guidelines for rare malignancies during pregnancy.
